# Rpb1 Sumoylation in Response to UV Radiation or Transcriptional Impairment in Yeast

**DOI:** 10.1371/journal.pone.0005267

**Published:** 2009-04-22

**Authors:** Xuefeng Chen, Baojin Ding, Danielle LeJeune, Christine Ruggiero, Shisheng Li

**Affiliations:** Department of Comparative Biomedical Sciences, School of Veterinary Medicine, Louisiana State University, Baton Rouge, Louisiana, United States of America; Oregon State University, United States of America

## Abstract

Covalent modifications of proteins by ubiquitin and the Small Ubiquitin-like MOdifier (SUMO) have been revealed to be involved in a plethora of cellular processes, including transcription, DNA repair and DNA damage responses. It has been well known that in response to DNA damage that blocks transcription elongation, Rpb1, the largest subunit of RNA polymerase II (Pol II), is ubiquitylated and subsequently degraded in mammalian and yeast cells. However, it is still an enigma regarding how Pol II responds to damaged DNA and conveys signal(s) for DNA damage-related cellular processes. We found that Rpb1 is also sumoylated in yeast cells upon UV radiation or impairment of transcription elongation, and this modification is independent of DNA damage checkpoint activation. Ubc9, an E2 SUMO conjugase, and Siz1, an E3 SUMO ligase, play important roles in Rpb1 sumoylation. K1487, which is located in the acidic linker region between the C-terminal domain and the globular domain of Rpb1, is the major sumoylation site. Rpb1 sumoylation is not affected by its ubiquitylation, and *vice versa*, indicating that the two processes do not crosstalk. Abolishment of Rpb1 sumoylation at K1487 does not affect transcription elongation or transcription coupled repair (TCR) of UV-induced DNA damage. However, deficiency in TCR enhances UV-induced Rpb1 sumoylation, presumably due to the persistence of transcription-blocking DNA lesions in the transcribed strand of a gene. Remarkably, abolishment of Rpb1 sumoylation at K1487 causes enhanced and prolonged UV-induced phosphorylation of Rad53, especially in TCR-deficient cells, suggesting that the sumoylation plays a role in restraining the DNA damage checkpoint response caused by transcription-blocking lesions. Our results demonstrate a novel covalent modification of Rpb1 in response to UV induced DNA damage or transcriptional impairment, and unravel an important link between the modification and the DNA damage checkpoint response.

## Introduction

The integrity of cellular DNA is constantly challenged by both endogenous and exogenous sources, including oxygen radicals within cells, environmental UV light, ionizing radiation and other genotoxic agents [1]. Maintenance of the fidelity of genetic material is critical for preserving normal cell function and preventing tumorigenesis of normal cells. To survive and generate viable progeny, cells must assess the damage and then either repair it or trigger the apoptotic program. A major component of the response is the DNA damage checkpoint, which arrests the cell cycle to provide time for carrying out DNA repair. In budding yeast, Mec1, the counterpart of mammalian ATR (Ataxia-Telangiectasia mutated and Rad3-related), and Tel1, the counterpart of mammalian ATM (Ataxia-Telangiectasia Mutated), are the kinases that sense DNA damage [2]. Mec1 is activated by long 3′-ended single stranded DNA (ssDNA) tails generated during resection of double strand breaks or by ssDNA gaps arising in repair. Tel1 is activated by unresected, blunt-ended DNA [2]. Rad53, the counterpart of the mammalian Chk2, is the major effector of the DNA damage checkpoint, and its phosphorylation by Mec1 has been considered a hallmark of checkpoint activation in yeast. Phosphorylated Rad53 targets a number of substrate proteins, resulting in stabilization of stalled replisomes, suppression of recombination, and prevention of cell cycle progression [2].

Multiple mechanisms have evolved to repair damaged DNA, including the versatile nucleotide excision repair (NER) which is capable of removing a variety of bulky helix-distorting lesions, such as UV-induced cyclobutane pyrimidine dimers (CPDs) and 6-4 photoproducts [3]. NER has been grouped into two pathways, *i.e*., global genomic repair (GGR) and transcription coupled repair (TCR). GGR is operative throughout the genome, and is dependent on XPC (xeroderma pigmentosum complemention group C) [4] in mammals or Rad7 and Rad16 in *S. cerevisiae* [5]. TCR, which is believed to be triggered by the stalling of RNA polymerase II (Pol II), is dedicated to rapid repair of the transcribed strand of actively transcribed genes [3]. In human cells, Cockayne's syndrome (CS) complementation group A and B (CSA and CSB) proteins are specifically required for TCR but dispensable for GGR [6], [7], [8]. In *S. cerevisiae*, Rad26, the homolog of human CSB [9], and Rpb9 [10], [11], a nonessential subunit of Pol II, have been shown to be specifically involved in TCR.

The fate of the stalled Pol II remains one of the major enigmas concerning how the cell reacts to damaged DNA [12]. Strikingly, in response to transcription-blocking DNA damage, Rpb1, the largest subunit of Pol II, is ubiquitylated and subsequently degraded [13], [14], [15], [16]. An earlier study in human cells showed that the TCR-specific proteins CSA and CSB are required for Rpb1 ubiquitylation and subsequent degradation, which led to the proposition that Pol II may need to be degraded for TCR to take place [14]. However, a recent report showed that the defects in Rpb1 ubiquitylation observed in CS cells are caused by an indirect mechanism: these cells shut down transcription in response to DNA damage, effectively depleting the substrate for ubiquitylation, namely elongating Pol II [17]. In yeast, several proteins, including Rsp5 [13], Elc1[18], Def1 [16] and Rpb9 [15], have been shown to be involved in Rpb1 ubiquitylation and subsequent degradation. However, Rsp5 [19], Elc1 [20] and Def1 [16] were shown to play no role in TCR. Interestingly, the domains of Rpb9 that are required for Rpb1 ubiquitylation are different from those that are involved in TCR [15], [21]. Together, the recent results indicate that Pol II ubiquitylation and degradation do not play a role in TCR.

The Small Ubiquitin-like MOdifier (SUMO) has been revealed to be involved in a plethora of cellular processes, including transcription, DNA repair, cell cycle progression, chromatin organization, nuclear transport, signal transduction and protein degradation [22], [23]. Like ubiquitin, SUMO is linked to its substrates via an amide bond between its C-terminal carboxyl group and the ε-amino group of a K residue in the substrate [22], [23]. SUMO modification in yeast is catalyzed by a three-step enzyme reaction, involving the heterodimeric activating enzyme (E1) Uba2/Aos1, the conjugating enzyme (E2) Ubc9, and a few ligases (E3). Sumoylation is a reversible process, and two specific isopeptidases, Ulp1 and Ulp2, were shown to be responsible for removing SUMO from modified proteins [24].

In this study, we identified SUMO as a novel covalent modification of Rpb1 in response to UV radiation or impairment of transcription elongation. We further characterized the sumoylation and found that it plays no role in transcription elongation or TCR and does not crosstalk with Rpb1 ubiquitylation. However, Rpb1 sumoylation was found to be affected by activities of NER, particularly TCR. Remarkably, the sumoylation appears to function in restraining the DNA damage checkpoint response caused by transcription-blocking lesions.

## Results

### Rpb1 is sumoylated in response to UV-induced DNA damage

**Figure 1:**
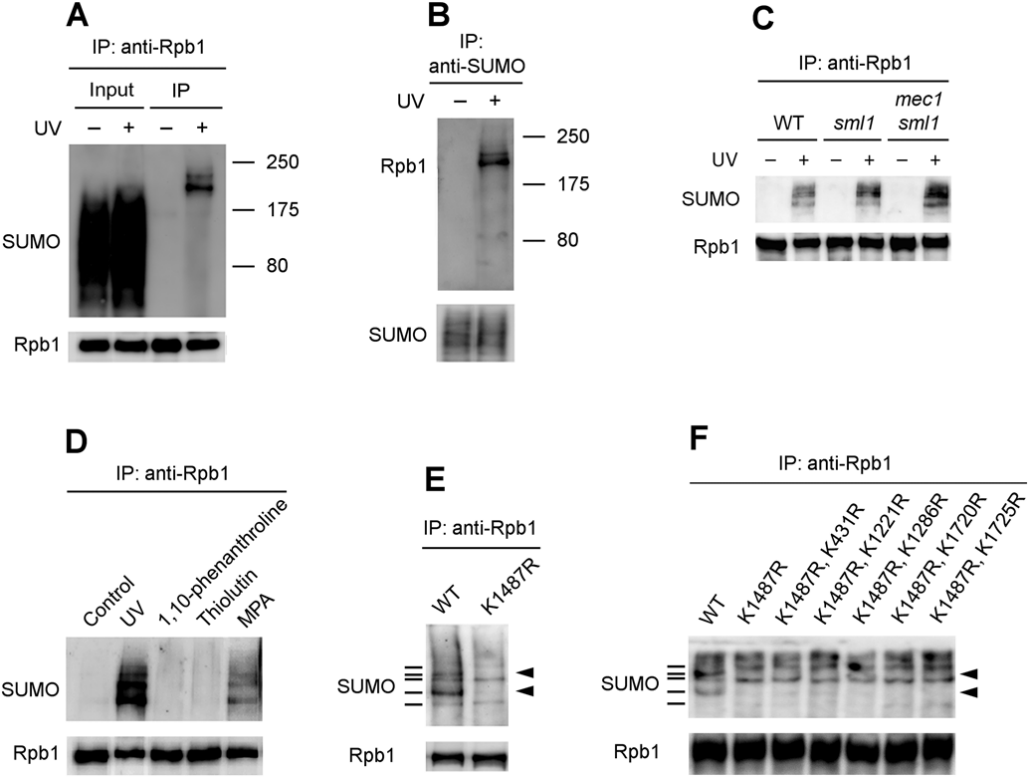
Western blots showing Rpb1 sumoylation in response to UV radiation or impairment of transcription elongation. (A) Rpb1 was immunoprecipitated from the unirradiated and UV irradiated cells using antibody 8WG16 (anti-Rpb1) and probed with anti-SUMO and 8WG16 antibodies. (B) Sumoylated proteins were immunoprecipitated from the unirradiated and UV irradiated cells and probed with 8WG16 and anti-SUMO antibodies. (C) UV-induced Rpb1 sumoylation in wild type (JKM179), *sml1* (YFD756) and *sml1 mec1* (YAA25) cells. (D) Sumoylation of Rpb1 in response to UV or treatments of transcription inhibitors. (E) UV-induced Rpb1 sumoylation in cells expressing wild type (CX84) or K1487R mutant (CX79) Rpb1. Bars on the left of the blot indicate distinct bands formed by wild type Rpb1. Arrow heads on the right of the blot mark bands abolished by the K1487R mutation. (F) UV-induced Rpb1 sumoylation in cells expressing wild type (CX84) or K to R mutant (CX79, CX105, CX106, CX108, CX110 and CX110) Rpb1. Bars on the left of the blot indicate distinct bands formed by wild type Rpb1. Arrow heads on the right of the blot mark bands not shown by the mutant Rpb1. WT, wild type.

TCR is believed to be triggered by the stalling of RNA polymerase II (Pol II) [3]. In response to UV-induced DNA damage, Rpb1, the largest subunit of Pol II, is ubiquitylated and subsequently degraded in both human and yeast cells [13], [14]. The early studies proposed that Pol II ubiquitylation and subsequent degradation may be required for TCR to take place. However, it was later found that these events are not related to TCR in either human [17] or yeast [15], [16], [19] cells. To explore potential Pol II-related signal(s) for TCR and/or other DNA damage responses, we examined other possible modifications of Rpb1 following UV irradiation. Rpb1 was immunoprecipited from yeast cells using antibody 8WG16 which specifically recognizes the C-terminal heptapeptide repeats of Rpb1 [25]. The immunoprecipitates were subject to Western blot and probed with antibodies that were known to recognize potential covalent modifications. Interestingly, when the immunoprecipitated Rpb1 was probed with an anti-SUMO antibody, several bands could be seen in the UV irradiated samples, but not in the unirradiated ones (Fig. 1 A), indicating that Rpb1 was sumoylated in response to UV-induced DNA damage. To confirm this finding, a reciprocal immunoprecipitation was carried out. Sumoylated proteins were immunoprecipitated from normally cultured and UV-irradiated cells using an anti-SUMO antibody, and the immunoprecipitates were probed with 8WG16 on a Western blot. Several bands could be detected in the UV irradiated sample, but not in the unirradiated one (Fig. 1B), indicating that Rpb1 is sumoylated in response to UV-induced DNA damage. The different bands may reflect different forms of sumoylated Rpb1 (e.g., mono-, poly- or multi-sumoylated).

### Activation of DNA damage checkpoint is not required for UV-induced Rpb1 sumoylation

DNA damage to a cell can activate checkpoint response, which promotes cell-cycle arrest, DNA repair, senescence or apoptosis [2], [26], [27]. To test if activation of DNA damage checkpoint is required for UV-induced Rpb1 sumoylation, we examined the modification in cells lacking Mec1, which plays a key role in activation of checkpoint in response to UV DNA damage [2]. Mec1 is essential for cell viability even in the absence of DNA damage [2]. The inviability of *mec1* cells is suppressed by increasing the activity of cellular ribonucleotide reductase (RNR) rather than by restoring DNA damage checkpoint function [28], [29], and the essential role of Mec1 during normal cell growth appears to be in stabilizing stalled replication forks [30], [31]. Simultaneously ablating Sml1, an inhibitor of the cellular RNR, restores the viability of *mec1* cells [32]. UV-induced Rpb1 sumoylation was slightly higher in *mec1 sml1* cells than in the isogenic wild type and *sml1* cells (Fig. 1C), indicating that this covalent modification is independent of the checkpoint activation. The slightly enhanced Rpb1 sumoylation in *mec1 sml1* cells is presumably due to the persistence (slower repair) of DNA damage in the absence of the checkpoint activation.

### Impairment of Pol II transcriptional elongation also induces Rpb1 sumoylation

UV-induced DNA lesions in the transcribed strand of a gene block Pol II transcription elongation [33]. We wondered whether Rpb1 sumoylation occurs specifically in response to UV-induced DNA damage or is due to blockage of Pol II transcription elongation. Several chemicals, such as mycophenolic acid (MPA), thiolutin and 1, 10-phenanthroline, have been used to inhibit transcription in yeast [34]. MPA inhibits transcription elongation by depleting cellular GTP pool, and sensitivity to this drug has been widely used as a landmark of transcription elongation deficiency [35]. Thiolutin inhibits transcription by all three RNA polymerases, mainly at the stage of transcription initiation [36]. 1, 10-phenanthroline is a metal chelator that most likely inhibits transcription by sequestering divalent metal ions [37]. Thiolutin and 1, 10-phenanthroline did not induce detectable Rpb1 sumoylation (Fig. 1D). However, MPA induced Rpb1 sumoylation to a certain level, which is lower than that induced by UV (Fig. 1D). These results suggest that Rpb1 sumoylation can be induced by impairment of transcription elongation. The reason that MPA induces a lower level of Rpb1 sumoylation than UV may reflect the fact that UV induced DNA damage may cause a more severe blockage of elongating Pol II.

### K1487 of Rpb1 is a major sumoylation site

We attempted to identify the site(s) of sumoylation on Rpb1. Sumoylation usually occurs on a lysine (K) residue located in the consensus motif ΨKxE/D (where Ψ is a hydrophobic residue and x is any residue) [23]. Rpb1 is a high molecular-weight protein (192 kD) with a total of 93 K residues. A sequence search indicated that K1487 is located in the sumoylation motif (VKDE). We created centromeric *LEU2* plasmids encoding the wild type and a mutant Rpb1 with an R replacing the K at site 1487 (K1487R). The *LEU2* plasmids were shuffled into yeast cells whose genomic *RPB1* gene was deleted. Yeast cells expressing the mutant Rpb1 grew normally under all conditions examined (not shown). Wild type and the mutant Rpb1 were immunoprecipitated from the respective cells following UV irradiation and probed with an anti-SUMO antibody on a Western blot. The K1487R mutation caused disappearance of a major and a minor band reflecting different forms of sumoylated Rpb1 (Fig. 1E). This indicates that K1487 is the major sumoylation site in response to UV-induced DNA damage.

Several Ks of Rpb1, namely K431 (WKVE), K1221 (FKND), K1286 (MKYD), K1720 (PKQD) and K1725 (QKHN) are located in sequences that are similar to the sumoylation motif. To test if these Ks are the minor sumoylation sites, we created plasmids encoding mutant Rpb1 with Rs replacing K1487 and each of these Ks. The additional K to R mutations did not change the pattern of UV induced Rpb1 sumoylation (Fig. 1F), indicating that these Ks (other than K1487) are not the minor sumoylation sites.

### Ubc9 and Siz1 play important roles in Rpb1 sumoylation

In *S. cerevisiae*, a single essential gene, *SMT3*, encodes the SUMO (Smt3) protein [23]. The SUMO is activated by an E1 activating enzyme and then passed to an E2 conjugase. An E3 ligase acts as an adapter to interact with both E2 and substrates and promote the transfer of SUMO from E2 to specific substrates [23].

**Figure 2:**
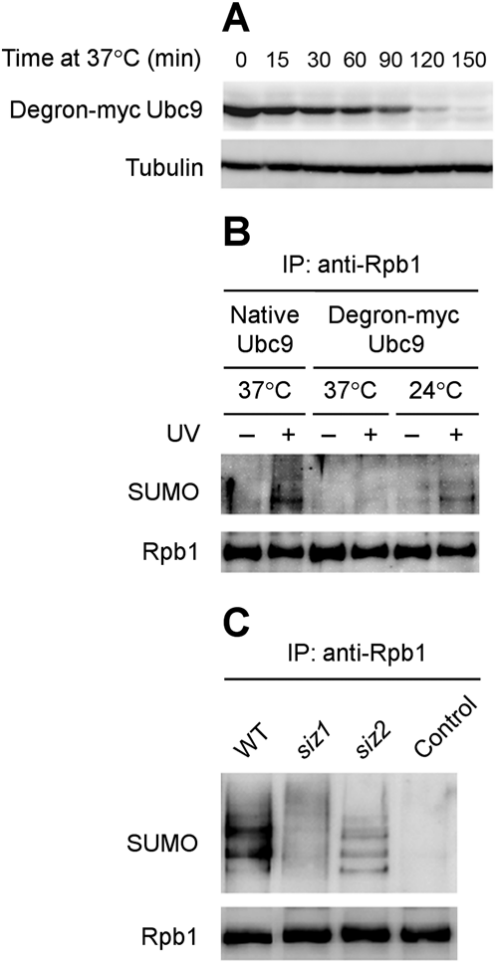
Western blots showing the roles of Ubc9 and Siz1 in UV-induced Rpb1 sumoylation. (A) Degradation of degron-myc tagged Ubc9 upon shifting to nonpermissive temperature (37°C) in galactose containing medium (to induce the expression of plasmid pKL142 encoded Ubr1, a ubiquitin E3 ligase). Tubulin serves as an internal loading control. (B) Abolishment of UV-induced Rpb1 sumoylation when Ubc9 was depleted. Rpb1 was immunoprecipitated from the cells cultured at the indicated conditions using antibody 8WG16 and probed with anti-SUMO and 8WG16 antibodies. (C) The roles of Siz1 and Siz2 in UV-induced Rpb1 sumoylation. Rpb1 was immunoprecipited from the UV irradiated wild type (BY4741) and mutant (strains 4245 and 2412) cells using antibody 8WG16 and probed with anti-SUMO and 8WG16 antibodies. The control was a sample prepared from unirradiated wild type cells. WT, wild type.

Ubc9, which is essential for cell viability, is the only SUMO E2 conjugase identified so far in yeast [23]. To examine if Ubc9 is involved in Rpb1 sumoylation, we inserted the degron-myc sequences [38], [39] in-frame at the 5′ end of the coding region of the genomic *UBC9* gene, thereby expressing the Ubc9 protein with a degron-myc tag at the N-terminus. Degron tagging has been successfully used to conditionally degrade an essential protein in yeast [38], [39]. A degron tagged protein can function normally in cells at permissive temperature (24°C), but is rapidly degraded through a ubiquitin-mediated pathway at non-permissive temperature (37°C) [39]. The myc tag following the degron tag is used for detection of a tagged protein with a generic anti-myc antibody [38], [39]. The tagged Ubc9 was degraded to an undetectable level ∼2.5 hours after the cells were shifted to 37°C (Fig. 2A). In cells expressing the degron-myc tagged Ubc9, UV induced Rpb1 sumoylation occurred normally at the permissive temperature but was undetectable at the non-permissive temperature (Fig. 2B). On the other hand, Rpb1 sumoylation occurred normally in cells expressing the native Ubc9 at the nonpermissive temperature. These results indicate that the SUMO E2 conjugase Ubc9 plays an important role in Rpb1 sumoylation.

In yeast, four SUMO E3 ligases have been identified: Siz1, Siz2 (Nfi1) [40], [41], Mms21/Nse2 [42], and Zip3 [43]. To identify the E3 ligase(s) that is/are involved in Rpb1 sumoylation, we started with testing Siz1 and Siz2, because they have been shown to be the major SUMO E3 ligases [40]. The prominent bands of sumoylated Rpb1 shown in wild type cells were essentially abolished in *siz1* cells (Fig. 2C), indicating that Siz1 plays an important role in Rpb1 sumoylation. However, some high molecular weight bands/smear of sumoylated Rpb1 appear to be somewhat enhanced in *siz1* cells (Fig. 2C, upper part of the blot), presumably reflecting a higher induction of highly sumoylated Rpb1 in the mutant cells.

The pattern of bands reflecting different forms of sumoylated Rpb1 was altered in *siz2* cells: the prominent slower migrating bands were fainter whereas the intensity of the fastest migrating band (at the bottom of the blot) was slightly increased (Fig. 2C, compare the *siz2* and WT lanes). This suggests that Siz2 may, to a certain extent, facilitate induction of poly-sumoylation of Rpb1.

### Sumoylation of Rpb1 does not affect its UV-induced degradation

Sumoylation takes place on lysine residues, which can also be modified by ubiquitylation. A growing body of evidence shows the existence of cross-talk between the two processes. Sumoylation of a substrate could stabilize the protein by antagonizing ubiquitylation [for a recent review see [44]]. Recently, it was found that sumoylation can also promote the degradation of the modified protein by facilitating its ubiquitylation [44]. As UV induces both sumoylation and ubiquitylation of Rpb1, we wondered if there is crosstalk between the two processes.

**Figure 3:**
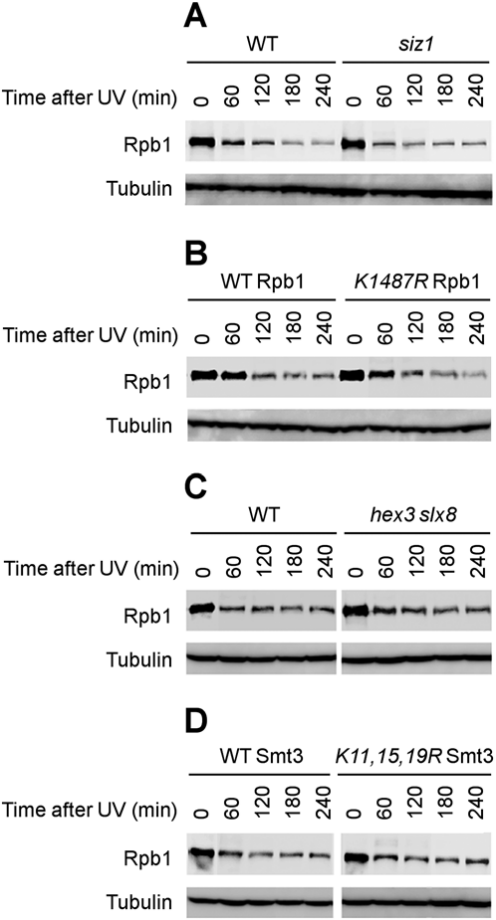
Sumoylation of Rpb1 does not affect its degradation in response to UV radiation. Whole cell extracts were prepared from the cells that had been incubated for different times following UV irradiation. Rpb1 in the whole cell extracts were probed with antibody 8WG16 on the Western blots. Tubulin serves as an internal loading control. (A) Levels of Rpb1 in isogenic wild type (BY4741) and *siz1* (strain 4245) cells. (B) Levels of wild type and K1487R mutant Rpb1 expressed in isogenic cells (CX84 and CX79). (C) Levels of Rpb1 in wild type (Y452) and *hex3 slx8* (MHY3861) cells. (D) Levels of Rpb1 in isogenic cells expressing wild type (JD74-13c) or K11,15,19R mutant Smt3 (YKU116). WT, wild type.

First, we examined if sumoylation of Rpb1 affects UV-induced Rpb1 degradation, which has been shown to be dependent on a prior ubiquitylation event [13], [15], [16]. UV induced Rpb1 degradation was not compromised in *siz1* cells (Fig. 3A), where Rpb1 sumoylation was virtually abolished (see above, Fig. 2C). Moreover, a K to R mutation at residue 1487, the major sumoylation site of Rpb1 (see above, Fig. 1E and F), does not cause any noticeable alteration in the UV induced Rpb1 degradation (Fig. 3B). These results indicate that UV-induced Rpb1 degradation, which is dependent on prior ubiquitylaiton, is not dependent on Rpb1 sumoylation.

Hex3 (Slx5) and Slx8 are yeast proteins with important functions in DNA damage control and maintenance of genomic stability [45], [46], [47], [48]. Several recent studies showed that the Hex3/Slx8 complex is an E3 ligase that specifically ubiquitylates sumoylated proteins in yeast [48], [49], [50]. Deficiency in the Hex3/Slx8 ubiquitin ligase causes the accumulation of sumoylated proteins [46]. To examine if the UV induced Rpb1 sumoylation is the substrate of the Hex3/Slx8 E3 ubiquitin ligase, we examined UV induced Rpb1 degradation in *hex3 slx8* cells and found that the degradation rate was similar to that in wild type cells (Fig 3C). Also, UV induced sumoylated Rpb1 did not accumulate in *hex3 slx8* cells (not shown), indicating that the sumoylated Rpb1 is not a substrate for the Hex3/Slx8 ubiquitin ligase.

A poly-SUMO chain is required for some sumoylated proteins to be targeted for ubiquitylation and subsequent degradation [51], [52]. Formation of poly-SUMO chains on a substrate needs K11, K15 or K19 of the Smt3 (SUMO) protein [53]. K to R mutations at all 3 sites block the formation of poly-SUMO chains but still allow mono-sumoylation on substrates [52]. We used cells expressing a mutant Smt3 whose K11, K15 and K19 were replaced by R residues to determine whether blockage of poly-sumoylation of Rpb1 affects Rpb1 degradation. Rpb1 was degraded normally in the mutant cells (Fig. 3D), indicating that the degradation is independent of Rpb1 poly-sumoylation. Taken together, our results strongly suggest that sumoylation of Rpb1 does not affect UV-induced Rpb1degradation. As Rpb1 degradation is dependent on a prior ubiquitylation event [13], [15], [16], it is unlikely that the sumoylation affects UV-induced Rpb1 ubiquitylation.

### Rpb1 ubiquitylation does not affect its sumoylation

**Figure 4:**
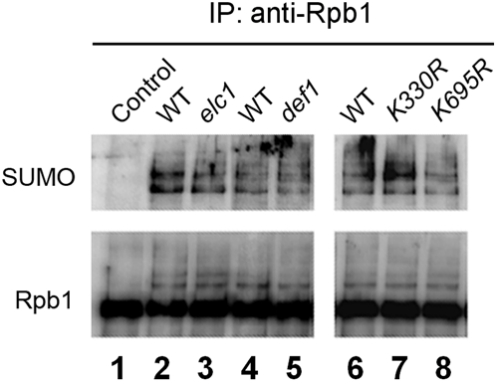
Ubiquitylation of Rpb1 does not affect its sumoylation. Rpb1 was immunoprecipitated from the unirradiated (control, BJ5465) and UV irradiated isogenic wild type [BJ5465 (lane 2) and Y452 (lane 4)], *elc1* (CR105) and *def1* (SL128) cells and cells expressing wild type [CX84 (lane 6)], K330R (CR191) or K695R (CR192) mutant Rpb1 using antibody 8WG16 and probed with anti-SUMO and 8WG16 antibodies.

We wondered if ubiquitylation of Rpb1 play a role in its sumoylation. Def1 [16] and Elc1 [18] are required for UV induced Rpb1 ubiquitylation and degradation in yeast. The patterns of Rpb1 sumoylation were similar between *def1* or *elc1* and their respective isogenic wild type cells (Fig. 4), indicating that Rpb1 ubiquitylation does not affect its sumoylation.

We also directly examined if blockage of Rpb1 ubiquitylation sites affects Rpb1 sumoylation. On Rpb1, K330 and K695 are the major and minor ubiquitylation sites, respectively [54]. While a K695R mutant Rpb1 was almost normally degraded upon UV irradiation [15], a K330R mutant Rpb1 was essentially not degraded following UV irradiation (not shown), in agreement with a previous report [54]. However, neither the K330R nor the K695R mutation affects the pattern of UV-induced Rpb1 sumoylation (Fig. 4). This indicates that the multiple Rpb1 bands detected by anti-SUMO antibody on the Western blots are not caused by concomitant ubiquityaltion, but may reflect different forms (mono-, poly- or multi-) of Rpb1 sumoylation. Taken together, our results suggest that Rpb1 ubiquitylation does not affect its sumoylation and *vice versa*: there is no apparent cross-talk between the two processes.

### Deficiency in TCR or entire NER enhances UV-induced Rpb1 sumoylation

**Figure 5:**
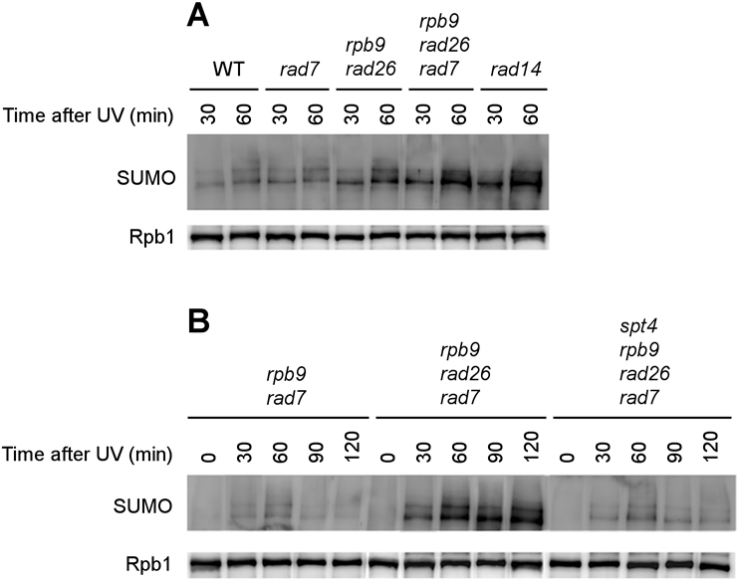
UV-induced sumoylation in wild type and NER-deficient cells. Log phase cells were irradiated with UV and incubated in a rich medium at 30°C. Rpb1 was immunoprecipitated from the cells at different times of the post-UV incubation using antibody 8WG16 and probed with anti-SUMO and 8WG16 antibodies. (A) UV-induced Rpb1 sumoylation in wild type (BJ5465), *rad7* (GGR-deficient) (SL212), *rad26 rpb9* (TCR-deficient) (SL81), *rad7 rad26 rpb9* (GGR- and TCR-deficient) (SL244) and *rad14* (GGR- and TCR-deficient) (CR14) cells. As Rpb1 was gradually degraded during the post-UV incubation in *RPB9* ^+^ (WT, *rad7* and *rad14*) cells [15], the loadings of samples from these cells at the different time points were adjusted to approximately the same level of Rpb1 remaining. (B) UV-induced Rpb1 sumoylation in *rad7 rpb9* (SL221), *rad7 rad26 rpb9* (SL244) and *rad7 rad26 rpb9 spt4* (SL243) cells.

Next, we examined if Rpb1 sumoylation interplays with NER, especially TCR. The UV-induced Rpb1 sumoylation was measured in wild type, *rad7* (GGR-deficient) [5], *rad26 rpb9* (TCR-deficient) [10], *rad7 rpb9 rad26* (TCR- and GGR-deficient) [10] and *rad14* (TCR- and GGR-deficient) [55] cells. UV-induced sumoylation of Rpb1 occurred in all of these strains (Fig. 5A), indicating that neither GGR nor TCR activity is essential for Rpb1 sumoylation. However, the levels of sumoylated Rpb1 were significantly higher in *rad26 rpb9*, *rad7 rpb9 rad26* and *rad14* cells than in wild type and *rad7* cells (Fig. 5A). These results indicate that deficiency in NER, particularly TCR, enhances the induction of Rpb1 sumoylation.

To confirm that the deficiency in TCR, rather than a specific repair factor, enhances the induction of Rpb1 sumoylation, we compared UV-induced Rpb1 sumoylations in *rad7 rpb9*, *rad7 rpb9 rad26* and *rad7 rpb9 rad26 spt4* cells. Deletion of the *SPT4* gene, which encodes a transcription elongation factor, partially restores TCR in *rad7 rad26* [56] and *rad7 rad26 rpb9* [21] cells. Interestingly, the level of UV-induced Rpb1 sumoylation in *rad7 rpb9 rad26 spt4* was similar to that in *rad7 rpb9* cells but significantly lower than that in *rad7 rpb9 rad26* cells (Fig. 5B), suggesting that the deficiency in TCR is the cause for the enhanced UV-induced Rpb1 sumoylation.

### Abolishment of Rpb1 sumoylation at K1487 does not affect TCR

**Figure 6:**
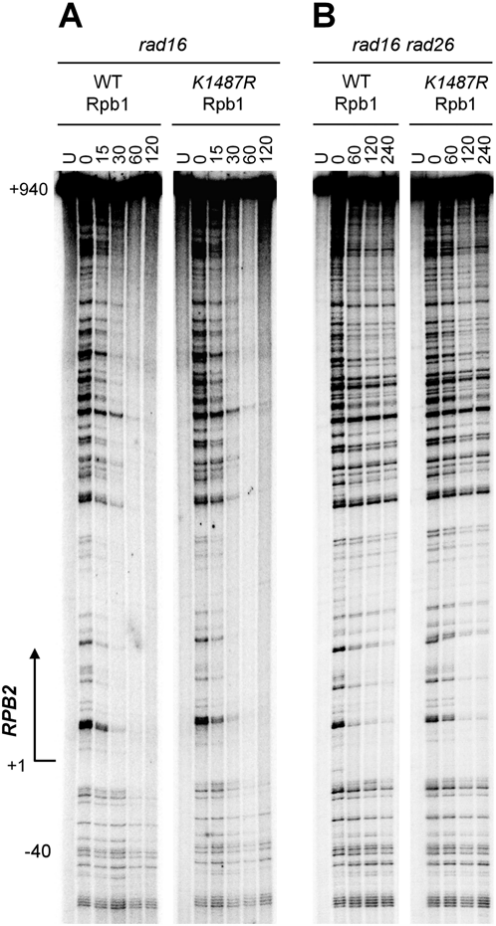
Abolishment of Rpb1 sumoylation at K1487 does not affect overall TCR or Rad26-independent TCR. (A) DNA sequencing gels showing repair of UV-induced cyclobutane pyrimidine dimers (CPDs) in the transcribed strand of the *RPB2* gene in *rad16* cells expressing wild type (CX85) or K1487R mutant (CX87) Rpb1. (B) DNA sequencing gels showing repair of CPDs in the transcribed strand of the *RPB2* gene in *rad16 rad26* cells expressing wild type (CX112) or K1487R mutant (CX113) Rpb1. Lanes *U* are unirradiated controls. Other lanes are samples from cells incubated for different times (min) following UV irradiation. The arrow on the left of the gels indicates the transcription start site of *RPB2*.

The observation that deficiency in TCR enhances UV-induced Rpb1 sumoylation can be explained by the persistence (or slower removal) of transcription-blocking lesions in the transcribed strand of the genes, as the mere impairment of transcription elongation by MPA treatment also induces Rpb1 sumoylation (Fig. 1D). Alternatively, Rpb1 sumoylation may serve as a TCR signal, which may be removed (Rpb1 de-sumoylated) during or after the TCR process in TCR-proficient cells. To test the second possibility, we examined the effect of K1487R mutation of Rpb1 on repair of CPDs in the constitutively transcribed *RPB2* gene in *rad16* cells where GGR is abolished [5], [57] and TCR can be unambiguously analyzed. Yeast cells were cultured to late log phase, UV irradiated, and incubated in a repair medium for various lengths of time. Total DNA was isolated, digested with a restriction enzyme to excise the fragment of interest, and incised at the UV-induced CPDs with an excess amount of T4 endonuclease V [58]. The incised fragments were strand-specifically end-labeled, resolved on a DNA sequencing gel, and exposed against a Phosphoimager screen. The band intensities in the gel lane of “0” time repair indicate the yields of CPDs at different sites. A decrease in band intensities with time at respective sites indicates CPD repair at these sites. In *rad16* cells expressing the wild type Rpb1, fast repair can be seen in the transcribed strand of the *RPB2* gene, initiating at ∼40 nucleotides upstream of the transcription start site (Fig. 6A), in agreement with our previous results [10]. The TCR rate in *rad16* cells expressing K1487R Rpb1 was similar to those expressing the wild-type Rpb1 (Fig. 6A). In agreement with previous results [9], [10], [11], [57], deletion of *RAD26* dramatically diminishes TCR in the *RPB2* gene (Fig. 6B). The TCR rate in *rad16 rad26* cells expressing K1487R Rpb1 was also similar to those expressing the wild-type Rpb1 (Fig. 6B). These results indicate that the K1487R mutation affects neither the overall TCR nor the Rad26-independent TCR.

In agreement with our TCR analysis results, mutating K1487 of Rpb1 to an arginine does not affect UV sensitivities of otherwise wild type, *rad16* and *rad16 rad26* cells (not shown). Also, yeast cells expressing K1487R mutant Rpb1 are not sensitive to the nucleotide depletion drug MPA (not shown), suggesting that the mutation does not significantly affect transcription elongation.

### Abolishment of Rpb1 sumoylation at K1487 enhances UV-induced Rad53 phosphorylation, especially in TCR-deficient cells

In human cell lines with defective TCR, stalled Pol II causes an increase in p53 levels and eventual induction of apoptosis [59]. Stalling of Pol II, caused by DNA damage, a DNA intercalating agent (actinomycin D) or microinjection of anti-Pol II antibodies in the nuclei, leads to p53 induction in a manner that depends on ATR and the single stranded DNA binding protein RPA [60], indicating that Pol II may function as a damage sensor for the DNA damage checkpoint response [60], [61]. Although *S. cerevisiae* lacks p53 and a long checkpoint arrest in G1 phase or a robust apoptotic pathway, the organism has a DNA damage checkpoint system that is similar to that in mammals [2]. In *S. cerevisiae*, phosphorylation of Rad53 (the human Chk2 homologue), an effector of DNA damage checkpoint, is an essential step for the cellular response to DNA damage and has been widely used as a marker for DNA damage checkpoint activation [2], [62], [63].

**Figure 7:**
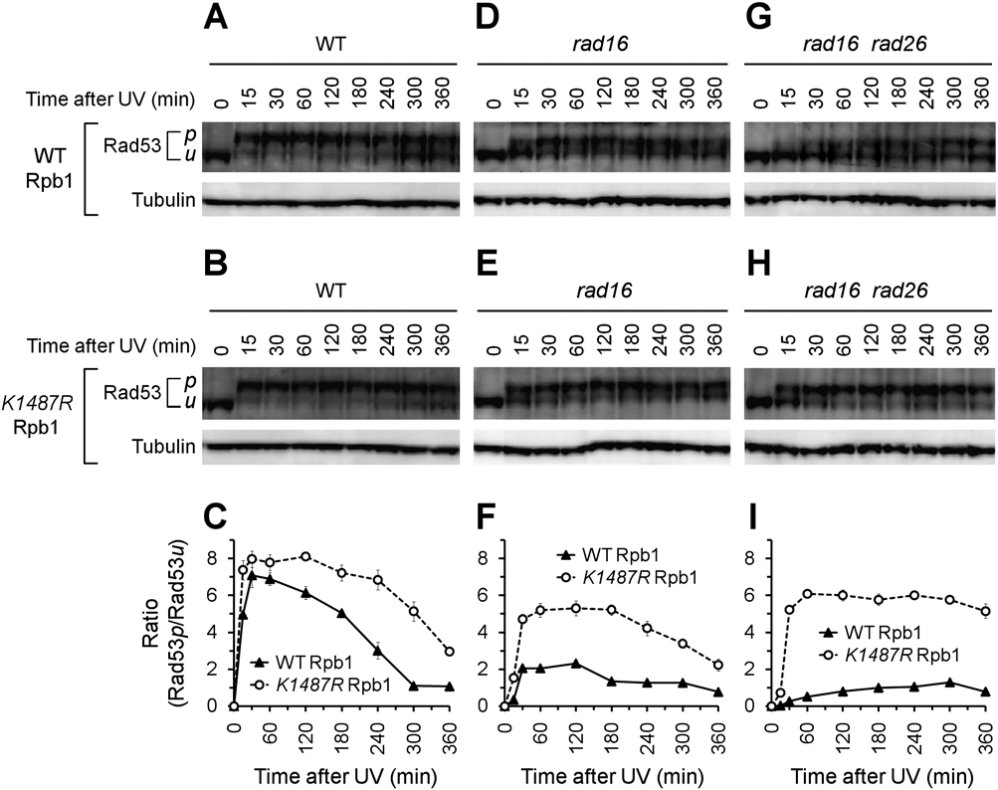
Effects of Rpb1 sumoylation at K1487 on UV-induced Rad53 phosphorylation. (A–C) UV-induced Rad53 phosphorylation in log phase wild type (for NER genes) cells expressing wild type (CX84) or K1487R mutant (CX79) Rpb1. (D–F) UV-induced Rad53 phosphorylation in log phase *rad16* cells expressing wild type (CX85) or K1487R mutant (CX87) Rpb1. (G–I) UV-induced Rad53 phosphorylation in log phase *rad16 rad26* cells expressing wild type (CX112) or K1487R mutant (CX113) Rpb1. The cells were irradiated with UV and incubated in a rich medium at 30°C. Whole cell extracts were prepared from the cells at different times of the post-UV incubation. Rad53 in the whole cell extracts was probed with an anti-Rad53 antibody on Western blots. *p* and *u* on the left of the blots indicate bands of phosphorylated and unphosphorylated Rad53, respectively. Plots C, F and I show ratios of phosphorylated Rad53 (Rad53*p*) to unphosphorylated Rad53 (Rad53*u*) in the wild type, *rad16* and *rad16 rad26* cells, respectively. Error bars represents standard deviations.

To examine if Rpb1 sumoylation plays a role in DNA damage checkpoint response, we analyzed UV-induced Rad53 phosphorylation in log phase yeast cells. In wild type cells, UV irradiation caused rapid phosphorylation of Rad53, which is reflected by the slower migrating Rad53 bands on a Western blot (Fig. 7A, see Rad53 bands marked with ‘*p*’). The level of Rad53 phosphorylation, as indicated by the ratio of the phosphorylated to unphophorylated Rad53, peaked ∼30 minutes after UV irradiation and gradually decreased afterwards (Fig. 7A and C). UV-induced Rad53 phosphorylation was somewhat weakened in *rad16* cells, and dramatically impaired and delayed in *rad16 rad26* cells (Fig. 7, compare panels A, D and G, and panels C, F and I). These results agree well with previous reports showing that both GGR mediated by Rad16 and TCR mediated by Rad26 contribute to DNA damage checkpoint response [62]. In fact, all yeast mutants deficient in incision during NER have been shown to be deficient in the rapid phosphorylation of Rad53 in response to UV radiation [3].

K1487R mutation of Rpb1 caused slightly enhanced and prolonged phosphorylation of Rad53 in otherwise wild type (for NER genes) cells in response to UV radiation (Fig. 7A, B and C). However, the K1487R mutation caused significant increase of UV-induced Rad53 phosphorylation in *rad16* (Fig. 7E and F) and *rad16 rad26* cells (Fig. 7H and I). Intriguingly, the K1487R mutation largely restored the rapid phosphorylation of Rad53 in *rad16 rad26* cells in response to UV radiation (Fig. 7H and I). We also examined the effects of the K1487R mutation on UV-induced Rad53 in cells synchronized at G1 (with α factor) and G2/M (with nocodazole) phases, and in stationary phase cultures. The general trends were similar to those obtained with the unsynchronized log phase cells (not shown). These results indicate that abolishment of Rpb1sumoylation at K1487 enhanced UV-induced Rad53 phosphorylation, especially in TCR deficient cells. In other words, sumoylation of Rpb1 at K1487 may play a role in restraining DNA damage checkpoint response caused by transcription-blocking lesions.

We also observed that treatment of wild type, *rad16* and *rad16 rad26* cells expressing wild type or the K1487R mutant Rpb1 with the nucleotide depleting drug MPA did not trigger phosphorylation of Rad53 (not shown), although the treatment triggers Rpb1 sumoylation (Fig. 1D). This indicates that mere impairment of transcription elongation may not be sufficient for inducing DNA damage checkpoint response in yeast.

## Discussion

In this study, we identified SUMO as a novel covalent modification of Rpb1 in response to UV DNA damage and impairment of transcription elongation, and unraveled an interesting connection between the modification and the DNA damage checkpoint response in yeast. Like ubiquitylation [64], sumoylation of Rpb1 can be induced by either UV radiation or nucleotide depletion drugs (*e.g.*, MPA), indicating that inductions of these modifications are not limited to DNA damage but are triggered by impairment of transcription elongation. However, Rpb1 ubiquitylation and sumoylation do not appear to have any crosstalk, as the K sites for the two modifications do not overlap and the events of UV-induced Rpb1 degradation (which is dependent on prior ubiquitylation) and sumoylation are mutually independent. Furthermore, Rpb1 sumoylation appears to be independent of DNA damage checkpoint activation, as the modification is not compromised in cells lacking Mec1 (Fig. 1C).

In many cases, without an E3 ligase, the E2 conjugase Ubc9 can directly attach SUMO protein to a substrate [23], [65]. The catalytic cleft of Ubc9 directly interacts with many substrates via their SUMO consensus motif (ψKxE/D), but this interaction is not sufficient for efficient SUMO transfer to the target K residue. Target modification therefore often depends on a third class of enzymes, the E3 ligases, which enhance SUMO transfer from the E2 to the substrate. Interestingly, our results indicate that while E3 ligase Siz1 is critical for Rpb1 sumoylation, the E3 ligase Siz2 may, to a certain extent, facilitate poly-sumoylation of Rpb1. These two E3 ligases may function competitively to achieve optimal sumoylation of Rpb1. In the absence of Siz1, more Rpb1 molecules may be available for Siz2 mediated sumoylation, which may result in the somewhat enhanced induction of high molecular weight forms of sumoylated Rpb1 in *siz1* cells (Fig. 2C, compare WT and *siz1* lanes). On the other hand, in the absence of Siz2, more Rpb1 may be available for Siz1, which may mainly mediate mono- or oligo-sumoylation of Rpb1.

TCR is generally believed to be initiated by Pol II stalled at a lesion in the transcribed strand of a gene [3]. However, the exact signal for TCR is still a mystery. Here, we present evidence that sumoylation at the major sumoylation site of Rpb1 (K1487) is not involved in TCR, as a K to R mutation at this site does not affect either the overall TCR or the Rad26-independent TCR (Fig. 6). Therefore, it is less likely that sumoylation of Rpb1 serves as a TCR signal. At present, however, we cannot rule out the possibility that sumoylation at some as-yet-unidentified minor site(s) of Rpb1 plays a role in TCR.

Our data show that sumoylation of Rpb1 is not dependent on TCR or the entire NER process, as the modification occurs in wild type and TCR- or NER-deficient cells (Fig. 5). However, Rpb1 sumoylation is enhanced in cells with deficiency in TCR or entire NER (Fig. 5). Our previous studies showed that Rpb1 ubiquitylation and subsequent degradation are also enhanced in TCR- or NER-deficient cells [15]. These enhancements are mostly likely due to the persistence (or slower removal) of Pol II-stalling lesions in the transcribed strand of a gene in these cells. This notion is supported by the findings that impairment of elongating Pol II by nucleotide depletion drugs also induces Rpb1 sumoylation (Fig. 1D) and ubiquitylation [64].

Single stranded DNA (ssDNA) is a useful common DNA damage checkpoint signal as it is formed during nucleotide and base excision repair, during resection at the ends of double stranded DNA breaks and at stalled replication forks [2]. It is generally accepted that the DNA damage checkpoint response can be triggered by interaction of ATR (or Mec1 in yeast) with RPA-coated ssDNA. A recent study showed that stalling of Pol II in mammalian cells, either by DNA damage or by non-DNA-damaging agents results in the phosphorylation of the serine 15 site of p53 in a RPA and ATR-dependent manner [60]. It was hypothesized that a region of ssDNA is formed after blockage of transcription elongation that attracts RPA, leading to the recruitment of ATR and the activation of p53 by phosphorylation of the serine 15 site. Consistent with this hypothesis is a study showing that RPA and ATR preferentially accumulate on transcribed DNA sequences after UV irradiation, presumably at sites of blocked RNA polymerases [66]. In this paper we show that abolishment of sumoylation of Rpb1 at K1487 enhanced UV-induced Rad53 phosphorylation, especially in TCR-deficient cells, establishing a link between Rpb1 sumoylation and checkpoint control. The molecular mechanism underlying the enhancement of Rad53 phosphorylation in cells expressing K1487R mutant Rpb1 remains to be elucidated. It is less likely that the enhancement is achieved by perturbation of transcription elongation or TCR, as the K1487R mutation does not affect either the sensitivity to the nucleotide depletion drug MPA (not shown) or TCR (Fig. 6). One possibility is that the abolishment of Rpb1 sumoylation at K1487 may alter the conformation of Pol II stalled at lesions, leading to generation or exposure of ssDNA regions that can trigger and/or sustain checkpoint response. Alternatively, sumoylated Rpb1 may recruit additional factors, which may in turn prevent the loading of a damage sensor to the ssDNA generated by damage-stalled Pol II.

What is the physiological function for Rpb1 sumoylation? It is believed that the major biological mission of the DNA damage checkpoint is to coordinate cellular processes allowing time to repair the damage so that the checkpoint-arrested cells can eventually resume cell cycle progression and continue their physiological program. Based on our results, one potential role for Rpb1 sumoylation may be to prevent spurious activation of checkpoint response by stalled Pol II. Interestingly, the K1487R mutation of Rpb1 does not appear to affect UV sensitivity of any cells tested, indicating that the role of Rpb1 sumoylation in the checkpoint response is not linked to cell survival.

Besides K1487, some other minor sumoylation sites on Rpb1 remain to be identified, and the role(s) of sumoylation at the other sites await to be determined. We observed that a mere impairment of transcription by MPA treatment did not cause Rad53 phosphorylation, although the treatment caused induction of Rpb1 sumoylation. In contrast, stalling of Pol II by some non-DNA-damaging agents induces DNA damage checkpoint activation in mammalian cells [53]. Therefore, it would be very interesting to test if Rpb1 sumoylation and the role of the modification are conserved between yeast and mammalian cells.

## Materials and Methods

### Yeast strains

The yeast strains used in the present work are listed in Table 1. To create gene deletion mutants, cells were transformed with linearized plasmids or PCR products bearing a selection marker (*URA3*, *LEU2* or *KanMX* genes) flanked by sequences complementary to the genes to be deleted. Strains expressing degron-myc tagged Ubc9 under the control of the copper-inducible promoter were created by transforming cells with PCR products generated using plasmid pKL187 as template [39]. Plasmid pKL142, which encodes Ubr1 (a ubquitin E3 ligase) under the control of the *GAL1* promoter, was transformed into the cells expressing the degron-myc tagged Ubc9 to promote rapid and conditional depletion of the degron tagged Ubc9 upon shifting to the nonpermissive temperature (37°C) in galactose containing media [39].

**Table 1:** Yeast strains used in this study.

Strain	Genotype^a^	Source/Reference
BJ5465	*MATa ura3-52 trp1 leu2Δ1 his3Δ200 pep4::HIS3 prb1Δ1.6R can1*	[72]
BY4741	*MATa his3Δ1 leu2Δ0 met15Δ0 ura3Δ0*	Open Biosystems
GHY498	*MATa his3Δ200 his4-912δ lys2-128δ leu2Δ1 ura3-52 rpb1Δ187::HIS3* [pRP112]	[73]
JD47-13c	*MATa leu2-Δ1 trp1-Δ63 his3-Δ200 ura3-52 lys2-801 ade2-101*	[52]
JKM179	*ho hml::ADE1 MATα hmr::ADE1 ade1-100 leu2-3,112 trp1::hisG lys5 ura3-52 ade3::GAL::HO*	[74]
MHY501	*MATα his3-Δ200 leu2-3,112 ura3-52 lys2-801 trp1-1 gal2*	[75]
Y452	*MATα ura3-52 his3-1 leu2-3 leu2-112*	L. Prakash
2412	as BY4741, but *Siz2::KanMX*	Open Biosystems
4245	as BY4741, but *Siz1::KanMX*	Open Biosystems
CR14	as BJ5465, but *rad14::URA3*	This study
CR105	as BJ5465, but *elc1::KanMX*	This study
CR109	as Y452, but *rad16::hisG degron-mycUBC9*	This study
CR191	as GHY498, but [pRP112] replaced with [ pJS670-K330R RPB1]	This study
CR192	as GHY498, but [pRP112] replaced with [ pJS670-K695R RPB1]	This study
CX79	as GHY498, but [pRP112] replaced with [ pJS670-K1487R RPB1]	This study
CX84	as GHY498, but [pRP112] replaced with [ pJS670]	This study
CX85	as Y452, but *rad16::hisG rpb1::KanMX* [ pJS670]	This study
CX87	as Y452, but *rad16::hisG rpb1::KanMX* [ pJS670-K1487R RPB1]	This study
CX105	as GHY498, but [pRP112] replaced with [ pJS670-K1487R K431R RPB1]	This study
CX106	as GHY498, but [pRP112] replaced with [ pJS670-K1487R K1221R RPB1]	This study
CX108	as GHY498, but [pRP112] replaced with [ pJS670-K1487R K1286R RPB1]	This study
CX110	as GHY498, but [pRP112] replaced with [ pJS670-K1487R K1720R RPB1]	This study
CX111	as GHY498, but [pRP112] replaced with [ pJS670-K1487R K1725R RPB1]	This study
CX112	as Y452, but *rad16::hisG rad26::URA rpb1::KanMX* [ pJS670]	This study
CX113	as Y452, but *rad16::hisG rad26::URA rpb1::KanMX* [ pJS670-K1487R RPB1]	This study
MHY3861	*hex3::KanMX4 slx8::KanMX4* (generated from a cross between the two single mutants)	[48]
SL81	as Y452, but *rad26::URA3 rpb9::LEU2*	[10]
SL128	as Y452, but *def1::URA3*	This study
SL212	as BJ5465, but *rad7Δ*	This study
SL221	as BJ5465, but *rad7Δ rpb9Δ*	This study
SL243	as BJ5465, but *rad7Δ rpb9Δ rad26::URA3 spt4:LEU2*	This study
SL244	as BJ5465, but *rad7Δ rpb9Δ rad26Δ*	This study
Y542-16	as Y452, but *rad16::hisG*	This study
YAA25	as JKM179, but *sml1::KAN mec1::NAT*	[74]
YFD756	as JKM179, but *sml1::KAN*	[74]
YKU116	as JD47-13c, but *Smt3-R11, 15, 19*	[52]

a Plasmid contained in a strain is shown in a bracket.

### Plasmid construction and shuffling

Plasmid pJS670, which bears the full length wild type *RPB1* gene on the centromeric *LEU2* vector pRS415 [67], was kindly provided by Dr. Jeff Strathern (the National Cancer Institute, NIH, Frederick, Maryland). Plasmids encoding Rpb1 with a K to R mutation at specific sites were created using plasmid pJS670 as template through site-directed mutagenesis (QuickChange II mutagenesis kit, Stratagene). The *LEU2* plasmids encoding the wild type or mutated Rpb1 were transformed into yeast strains whose genomic *RPB1* gene is deleted and complemented by a centromeric *URA3* plasmid encoding the wild type Rpb1. The *URA3* plasmid encoding the wild type Rpb1 was then evicted from the cells by selecting the transformed cells on plates containing 5-fluoroorotic acid (5-FOA), which will kill the cells with a functional *URA3* gene [68].

### Cell culture and UV irradiation

Unless otherwise indicated, yeast cells were grown at 30°C in minimal media containing glucose (SD) to late log phase (A_600_∼1.0), washed with ice-cold H_2_O and resuspended in ice-cold 2% glucose. For analyses of repair of UV-induced CPDs, the cell suspension was irradiated with 80 J/m^2^ of 254 nm UV. For analyses of UV-induced sumoylation and degradation of Rpb1 and phosphorylation of Rad53, the cell suspension was irradiated with 240 J/m^2^ of 254 nm UV. The irradiated cell suspension was added with one-tenth volume of a stock solution containing 10% yeast extract and 20% peptone and incubated in the dark at 30°C. Aliquots were removed from the cultures at different times of the incubation and the cells of the aliquots were harvested.

For analyses of the role of Ubc9 in UV-induced Rpb1 sumoylation, cells expressing native Ubc9 and those expressing degron-myc tagged Ubc9 and transformed with plasmid pKL142 were grown in minimal medium containing 2% raffinose and 1 mM of CuSO_4_ at the permissive temperature (24°C) to late log phase (A_600_∼1.0). Half of each of the cultures continued to be incubated at 24°C. The other half of each was washed with H_2_O, resuspended in pre-warmed (at 37°C) minimal medium containing galactose (SG) (without CuSO_4_) and incubated at 37°C for 2 hrs. The 24°C and 37°C cultures were irradiated with 240 J/m^2^ of 254 nm UV, and the cells were harvested after 1 hr of further incubation at the respective temperatures.

### NER analysis

Genomic DNA was isolated from the harvested cells that had been irradiated with 80 J/m^2^ of UV and incubated in the repair medium for different lengths of time, as described previously [10]. The gene fragments of interest were 3′-end labeled with [α-32P]dATP using a procedure described previously [69], [70]. Briefly, 1 µg of total genomic DNA was digested with restriction enzyme(s) to release the fragments of interest and incised at CPD sites with an excess amount of purified T4 endonuclease V (Epicentre). Excess copies of biotinylated oligonucleotides, which are complementary to the 3′ end of the fragments to be labeled, were mixed with the sample. The mixture was heated at 95°C for 5 min to denature the DNA and then cooled to an annealing temperature of around 50°C. The annealed fragments were attached to streptavidin-conjugated magnetic beads (Invitrogen), and the other fragments were removed by washing the beads at the annealing temperature. The attached fragments were labeled with [α-^32^P]dATP (Perkin-Elmer) and resolved on sequencing gels. The gels were dried and exposed against a Phosphorimager screen (Bio-Rad).

### Treatments of cells with transcription inhibitors

Yeast cells were grown in SD medium at 30°C to late log phase (A_600_∼1.0). 1, 10-phenanthroline, thiolutin and mycophenolic acid (MPA) were added to the cultures to final concentrations of 200 µg/ml, 10 µg/ml, and 200 µg/ml, respectively. After 1.5 hours of further incubation, the cells were harvested.

### Whole cell extract preparation

Whole cell extracts were prepared using a TCA method, as described previously [15]. Briefly, harvested cells were resuspended in 15% TCA and broken by vortexing them with acid-washed glass beads (Sigma, #G9268). The proteins in the cell lysates were pelleted by centrifugation at 20,000×*g* for 15 min. The protein pellet was washed with ice-cold 80% acetone, and dissolved in 2× SDS-PAGE gel loading buffer [71].

### Immunoprecipitation (IP)

Yeast cells harvested from 25 ml of culture were washed once with IP buffer (10 mM Tris-Cl, pH 7.4, 150 mM NaCl, 1 mM EDTA, 1 mM EGTA, 0.4 mM Na_4_VO_3_, 10 mM Na_4_P_2_O_7_, 10 mM NaF, 0.5% NP-40, 1% Triton X-100, 0.1% SDS, 0.2 mM PMSF and protease inhibitors cocktail) and resuspended in 0.5 ml of the same IP buffer. The cells were disrupted by vortexing with acid-washed glass beads and the cell lysates were cleared by centrifugation twice at 14,000 rpm for 5 minutes at 4°C. Eight µg of 8WG16 (Neoclone, WP011), which specifically recognizes the C-terminal heptapeptide repeats of Rpb1 [25], or anti-SUMO (Rockland, 200-401-428) antibody were added to the cell lysate and the mixture was incubated at 4°C overnight with gentle rotation. Protein A-coated agarose beads (Millipore) were added to the mixture and incubated at 4°C for 3 hours with gentle rotation. The beads were washed four times with IP buffer. Bound proteins were eluted by boiling the beads in 2× SDS-PAGE gel loading buffer [71].

### Western blot

Proteins in the whole cell extracts or immunoprecipitated samples were resolved on SDS-PAGE gels and transferred onto PVDF membranes (Immobilon-P, Millipore). Proteins of interest on the blots were probed with specific antibodies. The antibodies against the myc tag, tubulin and Rad53 were from Sigma (M4439), GeneTex (GTX76511) and Santa Cruz (sc-6749), respectively. Blots were incubated with SuperSignal® West Femto Maximum Sensitivity Substrate (Pierce), and the protein bands were detected using a chemiluminescence scanner (Fluorchem 8800, Alpha Innotech). As indicated, band intensities on some Western blots were quantified using AlphaEaseFC 4.0 software.
